# S-TOFHLA in mild Alzheimer’s disease and Mild Cognitive Impairment patients as a measure of functional literacy: Preliminary study

**DOI:** 10.1590/S1980-57642009DN30400005

**Published:** 2009

**Authors:** Maira Okada de Oliveira, Cláudia Sellitto Porto, Sonia Maria Dozzi Brucki

**Affiliations:** 1 Psychologist, Behavioral and Cognitive Neurology Unit, Department of Neurology of the University of São Paulo School of Medicine and Cognitive Disorders Reference Center (CEREDIC), Hospital das Clínicas of the University of São Paulo School of Medicine, São Paulo SP, Brazil.; 2 Psychologist, PhD, Behavioral and Cognitive Neurology Unit, Department of Neurology of the University of São Paulo School of Medicine and Cognitive Disorders Reference Center (CEREDIC), Hospital das Clínicas of the University of São Paulo School of Medicine, São Paulo SP, Brazil.; 3 MD, PhD, Behavioral and Cognitive Neurology Unit, Department of Neurology of the University of São Paulo School of Medicine and Cognitive Disorders Reference Center (CEREDIC), Hospital das Clínicas of the University of São Paulo School of Medicine, and Hospital Santa Marcelina, São Paulo SP, Brazil.

**Keywords:** Mild Cognitive Impairment, Alzheimer’s disease, S-TOFHLA, functional literacy

## Abstract

The greatest difficulty in diagnosing cognitive loss in our population is the diversity of its education which has a broad spectrum ranging from illiteracy, functional illiteracy and different degrees of literacy, even in those with the same level of schooling.

**Objectives:**

To verify whether there is impairment on the S-TOFHLA among individuals with AD and MCI compared with healthy controls, and to compare performance on the S-TOFHLA performance with neuropsychological tests and the scores achieved on the Raven’s Colored Matrices and Vocabulary and Block Design (WAIS-III) as a measure of estimated intellectual level.

**Methods:**

59 subjects: controls (n=23; age 70.96±8.31y; schooling 10.2±5.87y; 6 men), MCI patients (n=11; age 74.18±8.12y; schooling 7.55±4.32y; 5 men) and AD patients (n=25; age 76.16±4.96y; schooling 7.32±4.78y; 10 men) were submitted to neuropsychological assessment, S-TOFHLA and functional evaluation.

**Results:**

Differences on BD, Raven and Estimated IQ were found between controls and MCI patients as well as controls and AD patients. On the S-TOFHLA, differences were found between MCI and AD patients, controls and AD patients, but not between control and MCI groups. S-TOFHLA performance correlated strongly with schooling and all neuropsychological tests, except Clock Drawing.

**Conclusions:**

The S-TOFHLA seems to be a useful measure for determining the level of literacy in MCI patients, but not in AD patients. S-TOFHLA performance was more closely associated with neuropsychological test scores than were years of education and seems to be a good predictor of level of literacy. The Vocabulary subtest proved to be uninfluenced by the disease process in early stages and preserved in both MCI and AD patients, showing that semantic memory and crystallized intelligence are preserved.

## Abstract (pt)

A maior dificuldade no diagnóstico de perda cognitiva na nossa população é sua heterogeneidade educacional, com um espectro que vai do analfabetismo, analfabetismo funcional até os escolarizados com diferentes graus de alfabetização mesmo com o mesmo grau de escolarização.

**Objetivos:**

Verificar se existe comprometimento no S-TOFHLA em indivíduos com DA Leve e CCL comparados a controle saudáveis, correlacionando o desempenho do S-TOFHLA à avaliação neuropsicológica e aos escores alcançados no teste Matrizes Progressivas Coloridas de Raven e nos subtestes vocabulário e cubos (WAIS-III) como medida de nível intelectual estimado.

**Métodos:**

59 sujeitos controles (n=23; idade 70.96±8.31anos; escolaridade 10.2±5.87anos; 6 homens), pacientes com CCL (n=11; idade 74.18±8.12 anos; escolaridade 7.55±4.32anos; 5 homens) e pacientes com DA (n=25; idade 76.16±4.96; escolaridade 7.32±4.78;10 homens) foram submetidos a avaliação neuropsicológica, S-TOFHLA e avaliação funcional.

**Resultados:**

Em Cubos, Raven e QI Estimado foram encontradas diferenças estatísticas entre controle e pacientes com CCL; controles e pacientes com DA, mas não entre os grupos controle e CCL. O S-TOFHLA correlacionou fortemente com escolaridade e todos os testes neuropsicológicos, exceto Desenho do Relógio.

**Conclusões:**

O S-TOFHLA demonstrou ser uma medida útil em determinar o nível de alfabetismo em paciente com CCL, mas não em pacientes com DA. O S-TOFHLA apresentou maior foi mais relacionado com testes neuropsicológicos do que anos de escolaridade e parece ser um bom preditor do nível de alfabetismo. O subteste Vocabulário demonstrou ser um teste que não sofre com o processo da doença nos estágios iniciais não sofrendo alterações em pacientes com CCL e DA mostrando que memória semântica e inteligência cristalizada são preservadas.

Dementia is a syndrome characterized by the presence of cognitive decline that interferes in social or professional activities of individuals, representing a decline from previous levels.^1^ In Brazil, the prevalence of dementia is 7.1%, being higher in women and in elderly with lower educational level. The main cause of dementia is Alzheimer’s disease which is responsible for 55% of cases.^2^ The prevalence of cognitive and functional impairment in community-dwelling elderly subjects from the city of São Paulo is 16%.^3^

According to Petersen et al.,^4^ Mild Cognitive Impairment is a heterogeneous entity, which can be classified into: amnestic MCI single domain, amnestic MCI with multiple domains, nonamnestic MCI single domain or nonamnestic MCI multiple domains. Neuropsychological tests are widely recommended and used for diagnosing MCI. Boyle at al.,^5^ concluded that MCI is associated with a high risk of developing Alzheimer’s disease (AD) and reported that 25.8% of patients diagnosed with MCI developed AD after 2.5 years, a rate 6.7 times higher than those without cognitive impairment. However, these estimates vary from 4% to 40% per year.^6,7^ Despite the high rate of conversion to AD, some types of MCI appear to have higher risk, such as amnestic, single domain or multiple domains. However, a percentage of these patients will revert from cognitive impairment to normality.^8^

In the longitudinal study of Grober et al.,^9^ the elderly who developed dementia showed decline on tests of episodic memory, on average seven years prior to diagnosis, on tests of executive functions two to three years before the diagnosis of dementia while verbal intelligence quotient remained preserved up to approximately 0.4 years before diagnosis. Thus, the identification of individuals with potential risk of developing dementia becomes crucial.^10^

One difficulty in assessing cognitive decline is the low educational level of some patients, especially in developing countries like Brazil.

Illiteracy is a global problem that has been recognized and studied by many researchers. In Brazil, individuals who have less than four full years of schooling are considered functionally illiterate.^11^

Among the various definitions established, Barker^12^ presents functional health literacy as a set of individual capabilities that allows a person to acquire and use new information. These capabilities are by and large relatively stable but can improve with education programs or decline with aging or disease processes that affect cognitive functions.^13^ When individuals with functional illiteracy, or those who cannot read basic tasks required to function in society, or individuals who have low skills in reading, use the health system they have significant difficulties with routines for reading, such as reading prescriptions of vials of medication, consultation cards, instructions for personal care and education in health magazines.^14^ It is important to be able to identify people with limited ability to read, so they can be provided with special instructions on medications and chronic diseases. Basic skills in reading, writing and numbers are particularly important in the environment of health care where the patient’s participation in planning and implementing therapeutic regimens is essential for success. Adequate functional literacy means being able to apply these skills to health-related material such as prescription, consultation cards, labels of medications and instructions for health care at home.

Studies of functional literacy in health have been limited by the low number of instruments available for testing. Tools for assessing literacy such as the Wide Range Achievement Test - Revised (WRAT-R)^15^ can be used to determine the level of schooling, but interpreting the results is problematic because the level of education does not necessarily provide an accurate estimate of functional literacy in health.^16^ The most used instruments are the Rapid Estimate of Adult Literacy in Medicine (REALM), the Test of Functional Health Literacy in Adults (TOFHLA) and the short version of the latter test (S-TOFHLA). This test involves only health-related words, and has been used to identify a large proportion of ambulatory patients who have a poor ability to read. The REALM is a test containing 66 items of word recognition and pronunciation that measure the domain of vocabulary.^17^ However, this test does not measure the ability to read and understand numbers yet numeracy may be the most important element for functional health literacy.^16^

The TOFHLA was designed to measure patients’ ability to read and understand items commonly found in the health care setting using actual materials such as pill bottles and appointment slips.^16^ Although the original TOFHLA is an effective tool for identifying patients who have inadequate functional health literacy, it takes up to 20 minutes to administer. For this reason, the short TOFHLA (S-TOFHLA) was developed by reducing the TOFHLA to a version containing four Numeracy items and two prose passages. The S-TOFHLA has demonstrated similar reliability and validity to the full TOFHLA, but requires a maximum time of 12 minutes to complete.^14^ The S-TOFHLA is reliable and valid compared with REALM and helps to identify patients with inadequate functional health literacy.

Although the S-TOFHLA tests the reading ability of the patient, environmental health educators and professionals should be aware that individuals with functional illiteracy in health may also have difficulty understanding oral communication.^14^ Functional literacy is the ability to use reading, writing and computational skills at a level appropriate to meet the demands of routine situations. The TOFHLA is unique in that it measures functional health literacy in adults and is the most accurate indicator of reading ability of the patient because it measures the ability to read and understand the passages and numerical information.^14,16^ Moreover, TOFHLA and S-TOFHLA scores are an independent predictor of knowledge of the patient on chronic diseases and the ability to perform self-care, as well as on health status and use of health services.^14^

According to Barker et al.,^14^ there are significant differences between the S-TOFHLA and REALM. The Realm seems to both overestimate and underestimate the skill of reading in patients compared with the S-TOFHLA. Some patients are able to read individual words in the REALM and to pronounce them correctly, but fail to understand when reading is assessed using the S-TOFHLA. Conversely, some patients may have difficulty pronouncing isolated words on the REALM, but perform well on the S-TOFHLA when a context made available to help them.

In Brazil, a study was conducted involving 322 healthy subjects aged 20 to 92 years with education greater than or equal to a year, or informal education, and noted a strong correlation between number of years of schooling and scores achieved on the S-TOFHLA. Moreover, scores on the S-TOFHLA accounted for 49.3% of the variation in MMSE scores, correlating significantly and strongly with the MMSE, to a greater extent than the association of schooling.^18^

A high prevalence of AD and dementia has been found among elderly with low education. In a study by Manly et al.,^19^ the level of literacy was a better predictor of decline in memory, executive function and language than years of schooling. The result of the study showed that literacy should be regarded as a mediator of the interaction of biological and environmental factors regarding cognitive decline.

A previous study has shown that the ability to read is worse in elderly groups.^20^ Reading is a complex cognitive process that requires adequate vision, concentration, word recognition, operational memory and processing of information. Deficits in some of these areas can affect reading and understanding where the prevalence of these problems may increase with age.^13^

Neuropsychological tests are strongly influenced by education with practically all areas assessed yielding worse scores among illiterates and individuals with low education. Ardila and colleagues^21^ found that only immediate memory tests do not suffer from this influence. Several studies have evaluated this influence, including screening tests such as the MMSE,^22-24^ besides more extensive batteries such as the DRS,^25,26^ SKT^27^ and the ADAS-Cog.^28^ The main problem when using the number of years of formal education is that schools in Brazil have different programs depending on whether they are public or private, and also vary in the number of hours, and effectiveness of classroom learning. Consequently, we are faced with heterogeneous performance among elderly with the same level of education. Thus, a measure of the degree of literacy is urgently needed in order to support educational level in evaluating results of neuropsychological tests.

There are no studies using the S-TOFHLA as a measure of literacy in dementia. The S-TOFHLA is an instrument already in use among our population29and is relatively easy and quick to apply. It will be useful for determining the measure of level of literacy, which has been found to be more appropriate than the number of years schooling.

The goals of the present study were to verify whether there is impairment on the S-TOFHLA in individuals with AD and MCI compared to healthy controls, and to correlate the performance of the S-TOFHLA with neuropsychological assessment and schooling.

## Methods

The sample consisted of 59 subjects who were divided into three groups, containing controls (n=23), MCI patients (n=11) and AD patients (n=25) (Table 1).

**Table 1:** Demographic characteristics of control, MCI, and AD groups.

	Controls (n=23)			MCI (n=11)			AD (n=25)		p
	Mean	SD		Mean	SD		Mean	SD	
Gender (female/male)	17/6			6/5			15/10		0.45*
Age	70.96	±8.31		74.18	±8.12		76.16	±4.96	0.02**
Education	10.2	±5.87		7.55	±4.32		7.32	±4.78	0.18**

* Chi-Square Test;

** Kruskall-Wallis Test; SD, standard deviation; MCI, Mild Cognitive Impairment; AD, Alzheimer's disease.

The following general inclusion criteria were used for all participants: greater than one year of formal or informal education, more than 60 years old, presence of an informant, subject may be in use of stable-dose antidepressants for at least two months;

The following inclusion criteria were used for patients: absence of moderate or severe dementia, absence of dementia of other etiologies, absence of psychiatric disorders and absence of depressive symptoms according to the 15-item Geriatric Depression Scale (15-GDS)^30,31^ and Cornell Scale for Depression in Dementia.^32,33^

MCI patients were diagnosed based on the criteria proposed by Petersen et al.^4^ The diagnosis of probable AD was based on the National Institute of Neurological Disorders and Communicative Disorders and Stroke – Alzheimer’s Disease and Related Disorders Association (NINCDS-ADRDA) criteria.^34^ The patients had to be taking a maximum dose of anticholinesterasic. All patients were evaluated by staff members of the Behavioral and Cognitive Neurology Unit of the Department of Neurology at the University of São Paulo School of Medicine, from the Center of Cognitive Disorders (CEREDIC) and Hospital Santa Marcelina.

The following inclusion criteria were used for controls: absence of dementia according to the Brazilian version of the Mini-Mental State Exam (MMSE),^23^ absence of depressive symptoms according to the GDS-15 scale (score< 6)^30,31^ and the Cornell Scale for Depression in Dementia.^32,33^ (score< 7) and a score lower than two on the Functional Activities Questionnaire.^35^

### Cognitive evaluation

All subjects were submitted to the Brief Cognitive Screening Battery (BCSB)^36,37^ and Dementia Rating Scale (DRS), ^25,26^ which served as brief cognitive evaluations, and also to a comprehensive neuropsychological evaluation: Rey Auditory Verbal Learning Test (RAVLT),^38,39^ Raven’s Colored Matrices,^40^ Clock Drawing^41^ and Verbal Fluency.^38^

### Functional activities questionnaires

- Pfeffer Functional Activities Questionnaire.^35^
- Informant Questionnaire on Cognitive Decline in Elderly (IQCODE).^42-44^

### Literacy

- School Performance Test (SPT) – Reading subtest^45^: individuals have to read simple and complex words.

### Health literacy

S-TOFHLA: The reading comprehension text comprises two passages with a total of 36 items. The first text contains information about preparation for a gastrointestinal exam. The second is about rights and responsibilities of patients receiving health care in hospital. Each passage has every fifth or sixth word deleted and for each blank space, the respondent must select the word that best completes the sentence from a list of four words. The total score of the reading comprehension texts is 72 points, and every correctly filled blank space scores two points.

The numeracy test evaluates qualitative literacy needed in the health care setting. It comprises two medicine bottles and two cards containing information about medicine intake, date of appointments and the result of a laboratorial test. The numeric items total 28 points with seven points scored for each correct response. The total score of the test is 100 points. Individuals scoring between zero and 53 points are considered in the inadequate range; between 54 and 66 points, in the marginal range, and between 67 and 100, in the adequate range.

### Mood evaluation

- Cornell Scale for Depression in Dementia. ^32,33^
- 15-item Geriatric Depression Scale. ^30,31^

The study was approved by the Ethics Committee of the Hospital das Clínicas from the University of São Paulo School of Medicine and Hospital Santa Marcelina. All subjects who agreed to participate signed a written informed consent.

#### Statistical analysis

In order to evaluate associations between the categorical variables and the results, Pearson’s Chi-Squared test was performed. The Kruskall-Wallis test was used in analyses involving more than two samples. Spearman’s correlation analysis was applied to investigate association among tests. The value of significance accepted was 0.05.

All statistical analysis was carried out using the *Statistical Package for the Social Sciences* (SPSS) program, version 15.0.

## Results

No differences related to gender (p=0.45) or schooling (p=0.18) were found among the controls, MCI or AD patients, but a statistically significant difference for age was observed (p=0.02) (Table 1).

We also observed a significant difference among controls, MCI and AD patients which showed statistical significance on most tests except the DRS-Construction and Vocabulary (Table 2), and also among controls and MCI patients which revealed differences on general cognition (MMSE and DRS-Total), tasks related to memory (BCSB-Incidental memory, BCSB-Learning, BCSB-Delayed recall, DRS-Memory, RAVLT-Total and RAVLT-30’), to visuo-construction (Clock Drawing, Block Design) to visuo-perception, and fluid intelligence (Raven’s Colored Matrices) as well as the functional activities questionnaires (Pfeffer-FAQ and IQCODE). Comparison of controls and AD patients revealed statistical significance on most tests except Vocabulary and DRS-Construction. Differences were observed between MCI and AD patients on general cognition (MMSE and DRS-Total), on tasks of memory (BCSB-Immediate recall, BCSB-Learning, BCSB-Delayed recall, BCSB-Recognition, DRS-Memory, RAVLT-Total, RAVLT 30’), executive functions (Verbal Fluency animals, DRS-Initiation/Perseveration), educational level (S-TOFHLA, SPT-time) and functional activities questionnaires (FAQ and IQCODE).

**Table 2:** Performance of controls, aMCI and AD patients on the tests**.

Tests	Mean±SD			p
	Controls	aMCI	AD	
MMSE	28.35 (1.99)	26.73 (1.68)	24.24 (3.19)	<0.01
DRS – Attention	35.96 (1.11)	35.45 (1.44)	34.56 (1.19)	0.01
DRS – Initiation/Perseveration	35.13 (2.83)	34.27 (3.17)	30.36 (4.04)	<0.01
DRS – Construction	5.78 (0.42)	5.73 (0.65)	5.16 (1.31)	0.23
DRS – Conceptualization	36.87 (3.92)	37.09 (2.47)	32.60 (6.48)	0.02
DRS – Memory	23.52 (2.09)	19.55 (3.80)	15.80 (4.21)	<0.01
DRS – Total	137.26 (7.78)	132.09 (8.31)	118.48 (11.66)	<0.01
BCSB – Incidental memory	6.26 (1.60)	4.82 (1.54)	3.52 (1.76)	<0.01
BCSB – Immediate recall	7.87 (1.18)	7.09 (1.30)	5.52 (1.64)	<0.01
BCSB – Learning	8.91 (1.16)	7.64 (0.92)	5.88 (2.10)	<0.01
BCSB – Delayed recall	7.96 (1.15)	6.45 (1.37)	2.96 (2.54)	<0.01
BCSB – Recognition	9.87 (0.34)	9.64 (0.5)	7.44 (3.87)	<0.01
RAVLT – Total	42.35 (9.47)	29.55 (5.77)	22.56 (5.23)	<0.01
RAVLT – 30'	7.35 (3.95)	3.18 (1.83)	0.8 (1.38)	<0.01
Verbal Fluency (animals)	16.65 (5.94)	15.27 (2.61)	10.68 (4.07)	<0.01
Clock Drawing	9.00 (2.35)	7.27 (2.61)	6.80 (3.30)	0.02
IQCODE	3.09 (0.14)	3.49 (0.43)	4.07 (0.46)	<0.01
S-TOFHLA	72.53 (23.70)^§^	61.00 (18.78)^+^	40.56 (18.96)^+§^	<0.01
SPT (correct words)	68.35 (1.99)^§^	67.45 (2.42)	63.80 (7.56)	0.01
SPT (time- sec)	75.86 (22.88)^§^	86.45 (19.01)^+^	144.5(97.10)^+§^	<0.01
Vocabulary (WAIS-III)	30.35 (9.09)	27.09 (7.74)	24.80 (10.63)	0.22
Block Design (WAIS-III)	27.22 (8.26)*^§^	17.11 (7.69)*	15.14 (7.04)^§^	<0.01
Raven's Colored Matrices	27.09 (6.05)*^§^	22.36 (5.41)*	18.64 (5.37)^§^	<0.01
Estimated IQ	103.33 (9.19)*^§^	91.44 (8.76)*	88.95 (9.67)^§^	<0.01

** Kruskall-Wallis Test; SD, standard deviation; MCI, Mild Cognitive Impairment; AD, Alzheimer's disease; MMSE, Mini-Mental State Exam; BCSB, Brief Cognitive Screening Battery; DRS, Dementia Rating Scale; RAVLT, Rey Auditory Verbal Learning Test; IQCODE, Informant Questionnaire on Cognitive Decline in Elderly; S-TOFHLA, Short Test of Functional Health Literacy in Adults; SPT, School Performance Test; IQ, Intelligence Quotient; Kruskall-Wallis Test:

* Significant difference between control and MCI groups (p<0.005);

+ Significant difference between MCI and AD groups (p<0.005);

§ Significant difference between control and AD groups (p<0.005).

Comparison among control subjects, MCI and AD patients on S-TOFHLA, SPT, Vocabulary, Block Design, Raven’s Colored Matrices and Estimated IQ, revealed significant differences between all tests, except Vocabulary. The control and MCI groups had different performances on Block Design, Raven’s Colored Matrices and Estimated IQ. Among MCI and AD patients, statistically significant differences were found for S-TOFHLA and SPT-time, and comparing control group and AD patients found differences in S-TOFHLA, SPT (correct words), SPT (time-sec), Block Design, Raven’s Colored Matrices and Estimated IQ (Table 2).

Comparison among S-TOFHLA scores, schooling and neuropsychological tests showed that S-TOHLA correlated strongly with all neuropsychological tests, except Clock Drawing, and correlated negatively with SPT (time-sec) and IQCODE. Schooling correlated strongly with S-TOFHLA, MMSE, DRS-Total, RAVLT-Total, RAVLT-30’, Verbal Fluency, SPT (correct words), Vocabulary, Block Design, Raven’s Colored Matrices, Estimated IQ, and negatively with SPT (time-sec) (Table 3).

**Table 3:** Spearman's correlations among S-TOFHLA scores, schooling, and neuropsychological tests.

Neuropsychological tests	S-TOFHLAR	Schooling R
S-TOFHLA		0.564**
MMSE	0.701**	0.477**
DRS-Total	0.813**	0.519**
BCSB-Incidental memory	0.536**	
BCSB-Immediate recall	0.640**	0.268*
BCSB-Learning	0.493**	
BCSB-Delayed recall	0.570**	
BCSB-Recognition	0.546**	0.289*
RAVLT-Total	0.631**	0.441**
RAVLT – 30'	0.595**	0.373**
Verbal Fluency	0.636**	0.434**
Clock Drawing	0.297*	
SPT – (correct words)	0.705**	0.689**
SPT – (time -sec)	–0.716**	–0.459**
Vocabulary (WAIS-III)	0.625**	0.696**
Block Design (WAIS-III)	0.777**	0.514**
Raven's Colored Matrices	0.749**	0.558**
Estimated IQ	0.813**	0.747**
IQCODE	–0.597**	

* p<0.05;

** p<0.01;R coefficient of correlation; S-TOFHLA, Short Test of Functional Health Literacy in Adults; MMSE, Mini-Mental State Exam; DRS, Dementia Rating Scale; BCSB, Brief Cognitive Screening Battery; RAVLT, Rey Auditory Verbal Learning Test; SPT, School Performance Test; IQ, Intelligence Quotient; IQCODE, Informant Questionnaire on Cognitive Decline in Elderly.

## Discussion

Manly et al.,^46^ correctly observed that people 65 years of age and older had fewer opportunities to receive a formal education and that this lack of opportunity to attend school has more dramatic effects on literacy rates in less developed countries. In Brazil, the prevalence of illiteracy is 9.8% among adults and 32.2% among elderly people.^11^

Age and education are variables known to affect cognitive performance. Education can modulate age-related changes in cognition. Aging affects the speed of information processing and memory functions. Schooling provides the individual with a knowledge bank that can be retained to older ages, and education may also lead to more efficient learning strategies to continue to acquire information, even after completing formal schooling.^47^

In this study, comparison among controls, MCI and mild AD patients showed statistical significance in most tests, except DRS-Construction and Vocabulary. The DRS-Construction is considered to have low sensitivity to the effects of age and schooling.^48^ A subtest of the WAIS-III, Vocabulary, proved to be a test that was not influenced by the disease process and was preserved in controls, MCI patients and mild AD patients. This indicates that semantic memory and crystallized intelligence are preserved. Semantic memory and general knowledge about the world, both aspects of crystallized intelligence, are spared until much later in life.^49^

Different performances between control group and MCI patients as well as between controls group and AD patients, on tasks of visuo-perception, visuo-construction and fluid intelligence, seen with Raven’s Progressive Matrices, could be an indication of progressive compromise of fluid intelligence with disease progression.

The S-TOFHLA showed no statistically significant differences between controls and MCI patients, and seems to be a useful measure for determining the level of literacy in MCI patients, but not in AD patients.

The correlation among S-TOFHLA scores, schooling, and neuropsychological tests showed that S-TOFHLA correlates more strongly than schooling with all neuropsychological tests except Vocabulary, which had very similar correlation.

According to Manly et al.,^46^ schooling had no effect on delayed recall, but literacy level correlated with incidental memory and learning, despite no significant association with schooling. It seems that S-TOFHLA can be more sensitive to small differences in literacy and is a promising tool for assessing illiterate elders for dementia. In this study no correlation was detected between schooling and delayed recall.

In our study, the Clock Drawing Test showed no significant correlation with schooling, but did correlate with S-TOFHLA. On the other hand, in a recent study the researchers showed that the CDT is strongly influenced by educational level and appears to be inadequate for dementia screening in individuals with less than 5 years of formal education.^50^ The present results confirm our expectations that literacy level is more sensitive for detecting differences between subjects than is schooling, since our sample has a mean schooling of seven years.

Literacy level has a significant influence on the nature of performance on traditional neuropsychological measures of verbal and nonverbal skills. However, many studies were unable to distinguish between the effects of literacy and the effects of little or no exposure to formal education.^46^

The S-TOFHLA was more strongly correlated with the MMSE, DRS-Total, BCSB (incidental memory, immediate recall, learning, delayed recall, recognition), neuropsychological tests: RAVLT-Total, RAVLT–30’, Verbal Fluency, Clock Drawing, Block Design (WAIS-III), Raven’s Colored Matrices; literacy tests: SPT (correct words) and estimated IQ, than were years of education. The S-TOFHLA scale seems to be a good predictor of level of literacy and showed stability between controls and MCI, allowing its use to better evaluate patients with mild impairments in cognition. These are the first results comparing S-TOFHLA against neuropsychological tests showing these correlations.

A multivariate analysis should be performed on a larger number of patients in the final study, so as to reveal correlations without suffering the bias of confounding variables.
